# Hormonal and Behavioral Consequences of Social Isolation and Loneliness: Neuroendocrine Mechanisms and Clinical Implications

**DOI:** 10.3390/ijms27010084

**Published:** 2025-12-21

**Authors:** Volodymyr Mavrych, Ghaith K. Mansour, Ahmad W. Hajjar, Olena Bolgova

**Affiliations:** 1 College of Medicine, Alfaisal University, Riyadh 11533, Saudi Arabia; vmavrych@alfaisal.edu (V.M.); awhajjar@alfaisal.edu (A.W.H.); 2 College of Pharmacy, Alfaisal University, Riyadh 11533, Saudi Arabia; gkmansour@alfaisal.edu

**Keywords:** glucocorticoids, hypothalamo-hypophyseal system, loneliness, neuroendocrinology, neural pathways, pituitary-adrenal system, social isolation

## Abstract

Social isolation and loneliness represent critical psychosocial stressors associated with profound hormonal dysregulation and adverse behavioral outcomes. This review synthesizes current evidence on neuroendocrine mechanisms linking perceived and objective social disconnection to health consequences, emphasizing hypothalamic–pituitary–adrenal axis dysfunction, altered glucocorticoid signaling, and inflammatory pathways. Loneliness activates conserved transcriptional responses with upregulated proinflammatory gene expression and downregulated antiviral responses, mediated through sustained cortisol elevation and glucocorticoid resistance. Neural circuit alterations in reward processing, particularly the ventral tegmental area-nucleus accumbens pathway, contribute to anhedonia, social withdrawal, and cognitive decline. Sex differences in neuroendocrine responses reveal distinct hormonal profiles and circuit-specific adaptations. Emerging interventions targeting oxytocin and arginine vasopressin systems, alongside behavioral approaches addressing loneliness-induced cognitive biases, show promise. Critical research gaps include a mechanistic understanding of epigenetic modifications, sex-specific therapeutic responses, and translational applications across diverse populations. Understanding the endocrine–behavior interface in social disconnection offers opportunities for targeted interventions addressing this growing public health challenge.

## 1. Introduction

Social connection represents a fundamental biological need across mammalian species, with profound implications for survival, reproduction, and health [1]. The absence of meaningful social relationships manifests as either objective social isolation, defined by limited social contact, or subjective loneliness, characterized by perceived discordance between desired and actual social connections [2]. These conditions have reached epidemic proportions, affecting approximately one-quarter of older adults in developed nations, with prevalence rates of 33.9% documented among vulnerable populations [3]. Social isolation and loneliness exert mortality risks comparable to smoking and physical inactivity, necessitating urgent scientific attention [4].

The distinction between social isolation and loneliness carries important implications for understanding their biological consequences [1,2]. Social isolation refers to the objective lack of social contact and integration, measurable through frequency of interactions and social network size. Loneliness, conversely, represents the subjective distress arising from perceived inadequacy in quality or quantity of social relationships [2]. While correlated, these constructs demonstrate only moderate overlap (r = 0.3–0.5), and each predicts distinct health outcomes through partially overlapping yet mechanistically different pathways [5]. Both conditions activate stress-responsive neuroendocrine systems, yet their temporal dynamics and intensity differ substantially [6,7].

Historical perspectives on social disconnection trace back to early observations linking social environmental factors to physical health. Durkheim’s seminal work on social integration and mortality established foundational concepts linking social bonds to survival. Modern epidemiological research has quantified these relationships with precision, demonstrating that chronic loneliness increases mortality risk by 26%, comparable to well-established risk factors including obesity (23% increased risk) and physical inactivity (20–30% increased risk) [4]. Meta-analyses encompassing over 300,000 participants confirm robust associations between social disconnection and premature mortality across diverse populations and cultural contexts [4]. The prevalence of social isolation and loneliness has reached epidemic proportions, affecting approximately one-quarter of older adults in developed nations, with prevalence rates of 33.9% documented among vulnerable populations [3].

The neuroendocrine system serves as a primary transducer, converting psychosocial experiences into biological changes affecting health trajectories [8]. Social experiences modulate hormonal secretion patterns through neural circuits connecting higher-order social cognition regions with hypothalamic nuclei controlling endocrine function [9,10]. Chronic activation of these stress-responsive pathways produces allostatic load, the cumulative physiological wear resulting from repeated adaptation to stressors [11]. In the context of sustained social disconnection, allostatic load manifests as dysregulated cortisol rhythms, inflammatory activation, altered immune function, metabolic disturbances, and accelerated cellular aging [7,12]. The hypothalamic–pituitary–adrenal (HPA) axis represents the primary endocrine pathway mediating stress responses, with glucocorticoids serving as principal effector hormones that regulate physiological and behavioral adaptation [8]. Chronic activation of this system under conditions of sustained social disconnection produces maladaptive consequences, including immune dysregulation, metabolic dysfunction, and neuropsychiatric alterations [7].

Behavioral consequences of social isolation and loneliness extend beyond subjective distress to encompass cognitive, emotional, and motivational domains [13,14]. Longitudinal investigations demonstrate that loneliness predicts cognitive decline independent of objective social isolation, suggesting subjective perception drives neural and cognitive changes [15,16]. Neuroimaging studies reveal structural and functional brain alterations in regions supporting social cognition, emotional regulation, and reward processing [17,18,19]. These neural changes provide mechanistic substrates for the cognitive biases characteristic of chronic loneliness, including hypervigilance to social threats, negative interpretation of ambiguous social cues, and impaired ability to benefit from positive social interactions, a pattern that perpetuates social disconnection through maladaptive cognitive– behavioral cycles [13,14]. Recent advances in molecular neuroscience, optogenetics, and systems biology have illuminated specific neural circuits and gene expression patterns underlying loneliness-associated behavioral changes [20,21,22].

Recent advances in molecular neuroscience, optogenetics, and systems biology have illuminated specific neural circuits and gene expression patterns underlying loneliness-associated behavioral changes [20]. However, significant knowledge gaps persist regarding sex-specific mechanisms, developmental trajectories, and effective intervention strategies [23]. This review examines current understanding of hormonal and behavioral consequences of social isolation and loneliness, identifies controversies and research gaps, and discusses future directions for advancing this critical area of behavioral endocrinology.

## 2. Neuroendocrine Mechanisms of Social Isolation and Loneliness

### 2.1. HPA Axis Dysregulation and Glucocorticoid Signaling

Both objective social isolation and subjectively perceived loneliness activate the HPA axis through enhanced secretion of corticotropin-releasing hormone from hypothalamic neurons, stimulating adrenocorticotropic hormone release and subsequent glucocorticoid production, though evidence suggests stronger and more consistent HPA activation with chronic loneliness [9,11,24]. Acute social isolation triggers immediate cortisol elevation, serving adaptive functions by mobilizing energy resources and enhancing vigilance [24]. However, chronic loneliness produces sustained HPA axis activation with altered diurnal cortisol rhythms, characterized by flattened slopes, elevated awakening responses, and increased total daily cortisol output [11]. Longitudinal studies demonstrate that persistent loneliness correlates with hair cortisol concentrations, a biomarker of chronic HPA activation, with effect sizes comparable to major life stressors [25]. Critically, the relationship between loneliness and cortisol exhibits temporal specificity, with forced social isolation during pandemic lockdowns intensifying the positive association between momentary loneliness and salivary cortisol levels [6]. These findings suggest context-dependent modulation of neuroendocrine stress responses by social environmental factors [26].

The HPA axis normally operates through tightly regulated negative feedback mechanisms wherein cortisol binds to glucocorticoid receptors in the hippocampus, hypothalamus, and pituitary to suppress further CRH and ACTH release [27,28]. Under conditions of chronic loneliness, this feedback regulation becomes impaired, resulting in sustained cortisol elevation despite sufficient circulating levels to normally terminate the stress response [29]. This feedback dysregulation manifests across multiple time scales: blunted cortisol suppression following dexamethasone administration, delayed recovery to baseline following acute stressors, and loss of the normal circadian nadir in evening cortisol concentrations [30,31].

Paradoxically, chronic loneliness induces glucocorticoid resistance at the cellular level, wherein target tissues become less responsive to cortisol signaling despite elevated circulating levels [32]. This phenomenon involves altered expression and function of glucocorticoid receptors and their regulatory co-chaperones, particularly FK506 binding protein 5, which modulates receptor sensitivity [32]. Female rodents exposed to chronic adolescent stress exhibit elevated hippocampal glucocorticoid receptor-FKBP5 interactions following acute stress challenges in adulthood, potentially contributing to impaired stress recovery and increased vulnerability to affective disorders [32]. FKBP5 is itself a glucocorticoid-responsive gene that creates an intracellular ultra-short feedback loop: cortisol induces FKBP5 expression, which then attenuates glucocorticoid receptor activity, reducing the cell’s responsiveness to subsequent cortisol exposure [27,32]. Chronic loneliness may potentiate this cycle, leading to progressive glucocorticoid insensitivity in peripheral tissues, particularly immune cells, thereby disinhibiting inflammatory pathways despite elevated cortisol levels [28,32].

### 2.2. The Autonomic Nervous System and Catecholamine Signaling

Social isolation and loneliness produce profound autonomic nervous system dysregulation that contributes to both cardiovascular pathology and inflammatory activation [12,33]. The ANS represents a primary neuroendocrine regulatory system mediating the body’s rapid response to social stressors through sympathetic and parasympathetic branches [33].

Heart rate variability (HRV), an index of parasympathetic vagal tone and autonomic flexibility, demonstrates consistent reductions in lonely individuals [11,26]. Lower HRV reflects diminished parasympathetic buffering of sympathetic activation, creating conditions favoring inflammatory responses [33]. Electrodermal activity measurements reveal heightened sympathetic arousal during social evaluation paradigms among lonelier individuals, indicating sustained vigilance to social threat [27]. This chronic sympathetic predominance has cascading physiological consequences beyond cardiovascular function.

The sympathetic nervous system’s primary neurotransmitter, norepinephrine, serves as a critical mediator linking social disconnection to immune dysfunction [33]. Chronic sympathetic activation stimulates beta-adrenergic receptors (β2-AR) on immune cells, promoting proinflammatory polarization and myelopoiesis—the enhanced production of immune cells from bone marrow progenitors [33]. Norepinephrine, released from sympathetic nerve terminals innervating lymphoid organs and through systemic circulation during stress, binds to β2-adrenergic receptors on monocytes, macrophages, and dendritic cells [33]. This beta-adrenergic stimulation, in combination with impaired glucocorticoid signaling (Section 2.1), creates the molecular conditions for the conserved transcriptional response to adversity discussed in Section 2.3 [28,33].

Neuroimaging studies employing functional magnetic resonance imaging (fMRI) and electroencephalography (EEG) demonstrate that loneliness alters neural activation patterns that directly interface with autonomic control centers [13,17,18]. Lonely individuals show enhanced amygdala reactivity to negative social cues, coupled with reduced activation in reward-processing regions during positive social interactions [13,14,17]. These neural activation patterns correlate directly with sympathetic nervous system activity and inflammatory biomarkers, suggesting integrated psychoneuroimmunological pathways linking subjective social experience to physiological outcomes [27,31]. The amygdala’s projections to brainstem autonomic nuclei provide anatomical substrates for this mind–body connection.

### 2.3. Immune Dysregulation and Inflammatory Pathways

A cardinal feature of chronic loneliness involves proinflammatory immune activation mediated through neuroendocrine–immune interactions [27]. Lonelier individuals demonstrate enhanced synthesis of tumor necrosis factor-alpha (TNF-α) and interleukin-6 (IL-6) by peripheral blood mononuclear cells following acute stress exposure, independent of baseline inflammatory markers [27]. This heightened inflammatory reactivity reflects altered regulation of immune cell function by stress hormones [28].

Molecular analyses reveal that loneliness activates a conserved transcriptional response to adversity (CTRA), characterized by upregulated expression of proinflammatory genes and downregulated expression of genes involved in antiviral responses and antibody synthesis [29].

Critically, some studies demonstrated that CTRA gene expression correlates with UCLA Loneliness Scale scores (r = 0.21, *p* < 0.001) but shows no significant association with objective social network size when loneliness is statistically controlled, indicating that perceived social disconnection drives these transcriptional alterations [29].

**Figure 1:**
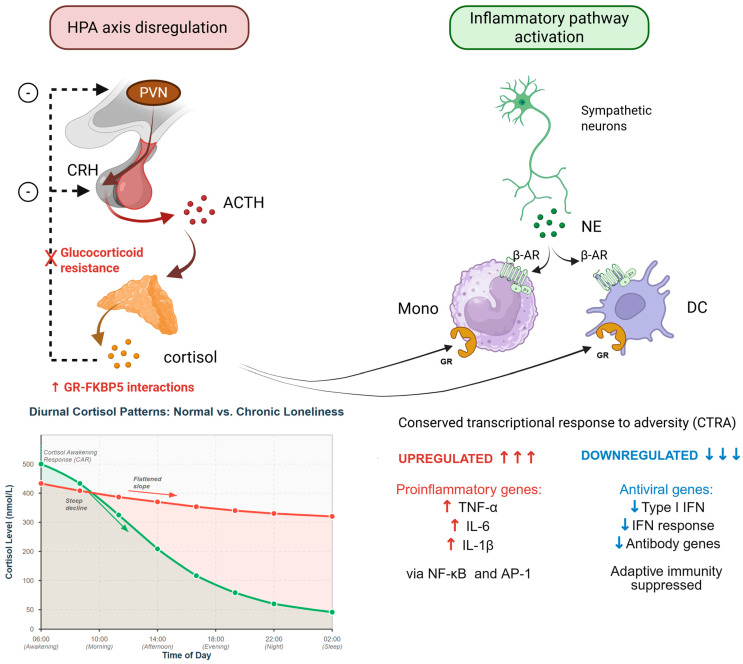
Neuroendocrine and Immune Mechanisms Linking Loneliness to Adverse Health Outcomes.

This transcriptional signature originates primarily from plasmacytoid dendritic cells and monocytes, with alterations in nuclear factor kappa B (NF-κB) and activator protein-1 (AP-1) transcription factors mediating gene expression changes [30]. The conserved transcriptional response to adversity represents a defensive biological strategy prioritizing innate immune responses to bacterial threats while suppressing adaptive immunity, an evolutionary adaptation to wounding risk during social conflict [28]. Mechanistically, glucocorticoid resistance contributes to sustained proinflammatory gene expression by impairing the anti-inflammatory actions of cortisol [31]. Additionally, loneliness enhances sympathetic nervous system activity, releasing norepinephrine that stimulates beta-adrenergic receptors on immune cells, further promoting proinflammatory cytokine production [33]. This immunometabolic dysregulation links loneliness to cardiovascular disease, metabolic syndrome, and accelerated cellular senescence [12]. Figure 1 illustrates the integrated neuroendocrine and immune pathways through which chronic loneliness produces systemic dysregulation.

- **HPA axis dysregulation:** HPA axis cascade shows corticotropin-releasing hormone (CRH) release from the hypothalamic paraventricular nucleus (PVN), stimulating adrenocorticotropic hormone (ACTH) secretion from the pituitary gland, which triggers cortisol release from the adrenal cortex.
- Graph inset depicts diurnal cortisol patterns: normal circadian rhythm (green curve) shows high morning levels with steep decline throughout the day, while chronic loneliness produces a flattened pattern (red curve) with reduced diurnal variation, elevated awakening response, and increased total daily output.
- The negative feedback loop (dashed black arrow) from cortisol to the hypothalamus is impaired (indicated by red X), representing glucocorticoid resistance. Glucocorticoid resistance involves increased GR-FKBP5 interactions that reduce receptor sensitivity and impair cortisol’s anti-inflammatory actions.
- **Inflammatory pathway activation:** sympathetic neuron releases norepinephrine (NE) that binds β-adrenergic receptors (β-AR) on peripheral immune cells, including monocytes and plasmacytoid dendritic cells. These cells also express glucocorticoid receptors (GR). The combination of β-adrenergic stimulation and impaired glucocorticoid signaling produces the conserved transcriptional response to adversity (CTRA). CTRA is characterized by upregulated expression of proinflammatory genes (TNF-α, IL-6, and IL-1β), via NF-κB and AP-1 transcription factors, and downregulated antiviral genes (type I interferons INF, antibody genes), suppressing adaptive immunity.

Created in BioRender. Mavrych, V. (2025) https://biorender.com/kv2nrdw.

Cellular immune changes in loneliness extend beyond cytokine production to encompass alterations in immune cell populations and their functional capacities [27,28,29]. T cell populations show altered distribution, with some studies reporting increased CD8+ cytotoxic T cells and decreased CD4+ helper T cell proportions [28,29]. T cell proliferative responses to mitogens demonstrate blunting in chronically lonely individuals, suggesting impaired adaptive immune responsiveness despite enhanced innate inflammatory activation [27,29]. This pattern aligns with the conserved transcriptional response to adversity, which prioritizes innate immunity while suppressing adaptive responses [28,29,30].

B-cell function, responsible for antibody production, shows impairment under chronic psychosocial stress conditions [29,30]. Reduced immunoglobulin responses to vaccination demonstrate functional consequences of this suppression [29]. Natural killer (NK) cell cytotoxicity, an important defense against viral infections and malignant cells, exhibits reduction in lonely individuals, potentially contributing to increased infection susceptibility and cancer risk [28,30].

General inflammatory biomarkers provide clinically accessible indices of systemic inflammation [12,27]. C-reactive protein (CRP), an acute-phase reactant synthesized by hepatocytes in response to interleukin-6, consistently demonstrates elevation in lonely individuals across multiple studies [12,27]. Erythrocyte sedimentation rate (ESR), another inflammatory marker, shows similar patterns [27]. Immunoglobulin levels (IgA, IgG, IgM) exhibit complex patterns, with some studies reporting elevations suggesting chronic immune activation, while others find reductions indicating immunosuppression—differences potentially reflecting measurement timing relative to acute versus chronic stress phases [29,30].

### 2.4. Neuropeptide Systems in Social Behavior Regulation

Oxytocin and arginine vasopressin, synthesized in hypothalamic paraventricular and supraoptic nuclei, represent critical neuropeptides regulating social behavior and stress responses [10]. Social isolation alters oxytocinergic signaling within mesocorticolimbic circuits, with reduced oxytocin receptor binding in regions mediating social reward, including the nucleus accumbens and prefrontal cortex [34]. Optogenetic studies demonstrate that oxytocin neurons in the paraventricular nucleus mediate social isolation-induced behavioral deficits through projections to the VTA [22].

Specifically, acute social isolation hyperactivates VTA dopamine neurons projecting to the prefrontal cortex, producing long-lasting synaptic plasticity characterized by insertion of GluA2-lacking AMPA receptors [21]. Chemogenetic inhibition of paraventricular oxytocin neurons reverses these cellular adaptations and rescues social preference deficits, establishing oxytocin regulation of dopamine circuits as necessary for maintaining normal social motivation [21]. Conversely, chemogenetic activation of oxytocin neurons recapitulates isolation-induced social withdrawal in group-housed animals [20]. Vasopressin signaling exhibits complementary roles in mediating social isolation responses, particularly through V1a and V1b receptor subtypes [2]. Loneliness correlates with reduced vasopressin reactivity, potentially contributing to diminished social attention and impaired navigation of social opportunities [2]. Intranasal oxytocin administration shows promise for reducing loneliness through enhanced oxytocinergic signaling, though individual variation in baseline bonding behaviors moderates treatment responsiveness [22].

### 2.5. Other Modulatory Factors

Beyond the major neuroendocrine axes described above, several additional systems modulate responses to social isolation.

#### 2.5.1. Neurotransmitter Systems

Neurotransmitter systems represent critical modulators of immune function through direct effects on immune cells expressing neurotransmitter receptors [31,33]. Serotonergic signaling, traditionally associated with mood regulation, demonstrates immunomodulatory properties through effects on cytokine production by T cells and macrophages [31]. Social isolation produces alterations in serotonin metabolism, with reduced serotonin availability contributing to both depressive symptoms and enhanced inflammatory responses [35,36]. Selective serotonin reuptake inhibitors demonstrate anti-inflammatory effects beyond their antidepressant actions, partially through restoration of serotonergic inhibition of proinflammatory cytokine production [31].

Dopaminergic neurotransmission interfaces with immune function through multiple mechanisms [1,21,35]. Dopamine receptors expressed on lymphocytes and monocytes mediate direct immunomodulatory effects, with dopamine generally exhibiting anti-inflammatory properties at physiological concentrations [31,35]. However, chronic stress-induced dopamine dysregulation may impair these protective effects [21,35]. Norepinephrine, released from sympathetic nerve terminals innervating lymphoid organs and through systemic circulation during stress, stimulates beta-2 adrenergic receptors on immune cells, promoting proinflammatory polarization [33].

GABAergic and glutamatergic signaling contribute to neuroimmune interactions through effects on microglial activation and blood–brain barrier integrity [21,36]. Chronic stress alters the balance between excitatory and inhibitory neurotransmission, potentially contributing to neuroinflammation observable in neuroimaging studies of lonely individuals [18,37].

#### 2.5.2. Endocrine Mediators Beyond Cortisol

While glucocorticoids represent primary stress hormones, additional endocrine factors contribute to loneliness-associated physiological dysregulation [8,9]. Sex hormones demonstrate bidirectional interactions with immune function, explaining partial sex differences in inflammatory responses to social isolation [32,38,39,40]. Estrogen exhibits generally anti-inflammatory properties through effects on nuclear factor kappa B signaling, while testosterone shows complex dose-dependent effects on immune function [38,40]. Fluctuations in progesterone during social stress may contribute to sex-specific coping responses and inflammatory profiles [38,40].

Thyroid hormones, regulated through the hypothalamic–pituitary–thyroid axis, demonstrate alterations under conditions of chronic psychosocial stress [8]. Thyroid-stimulating hormone (TSH), triiodothyronine (T3), and thyroxine (T4) influence metabolic rate, cardiovascular function, and immune activity [8]. Subclinical hypothyroidism associates with loneliness in older adults, potentially contributing to fatigue, cognitive slowing, and reduced social engagement [11,26].

Sleep-related hormones, including melatonin and orexin/hypocretin, demonstrate disruption under conditions of chronic loneliness [6,26,41]. Melatonin, secreted by the pineal gland in response to darkness, exhibits immunomodulatory and anti-inflammatory properties beyond its circadian regulatory functions [12]. Loneliness-associated sleep disturbances reduce melatonin secretion, potentially contributing to inflammatory activation [6,26].

## 3. Neural Circuits Underlying Loneliness-Associated Behaviors

### 3.1. Reward Circuitry Alterations

**Figure 2:**
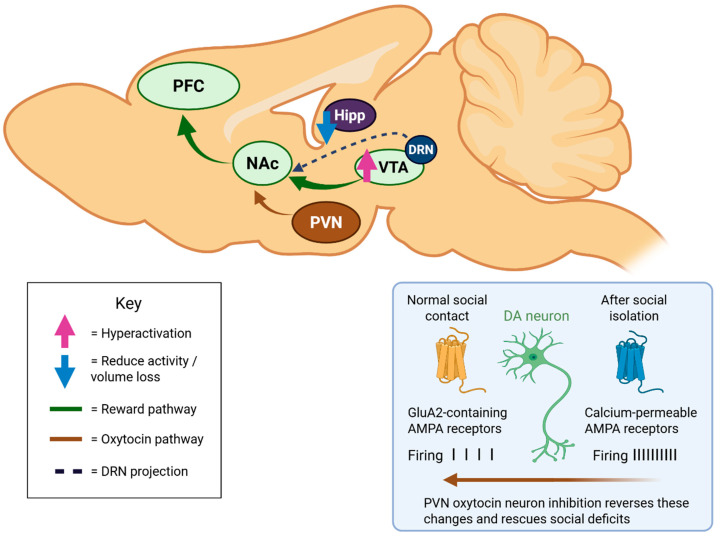
Neural Circuit Dysregulation in Chronic Loneliness.

Loneliness profoundly affects mesocorticolimbic reward circuits comprising the ventral tegmental area, nucleus accumbens, and prefrontal cortex [17]. Functional magnetic resonance imaging studies reveal that lonelier individuals exhibit increased ventral striatal activation when viewing close others, suggesting heightened reward value attribution to social reconnection opportunities [17]. This “yearning for connection” reflects adaptive motivation to restore social bonds, yet chronic loneliness paradoxically impairs social approach behaviors through maladaptive cognitive processes [13]. Social isolation in rodent models induces hyperexcitability of ventral tegmental area dopamine neurons, accompanied by altered firing patterns and neurotransmitter release dynamics [1]. These neurophysiological changes correlate with increased craving for social interaction, operationalized as conditioned place preference for social environments [20]. Notably, dorsal raphe dopamine neurons, distinct from classical ventral tegmental area populations, specifically encode loneliness-like states and causally drive social approach following isolation [35]. Figure 2 depicts these key neural circuits, including the cellular mechanisms of VTA dopamine neuron hyperexcitability and the critical role of paraventricular oxytocin neurons in modulating social motivation.

- **Sagittal view of brain regions** affected by social isolation and loneliness. Mesocorticolimbic reward circuit (green): VTA dopamine neurons show hyperactivation (↑) projecting to NAc and PFC, creating paradoxical increased social craving coupled with impaired social approach behaviors. Oxytocin regulatory pathway (brown): PVN oxytocin neurons project to VTA and modulate dopamine activity; chemogenetic inhibition of this pathway reverses isolation-induced deficits. Hippocampus (purple): Progressive gray matter volume reduction (↓) contributes to cognitive decline and dementia risk. Dorsal raphe dopamine neurons (blue dashed): DRN DA neurons, distinct from VTA populations, specifically encode loneliness states and drive social approach.
- **Inset: VTA dopamine neuron cellular adaptations**. Left: Normal social contact with GluA2-containing AMPA receptors. Right: After isolation, insertion of GluA2-lacking, calcium-permeable AMPA receptors produces hyperexcitability (↑) and long-lasting synaptic plasticity. PVN oxytocin neuron inhibition reverses these changes and rescues social deficits.

Created in BioRender. Mavrych, V. (2025) https://BioRender.com/2r68xr0.

### 3.2. Default Network and Limbic System Modifications

Large-scale neuroimaging studies reveal that loneliness is associated with altered structural and functional connectivity within the default mode network, comprising the medial prefrontal cortex, posterior cingulate cortex, and hippocampal formation [18]. Specifically, lonelier individuals exhibit reduced gray matter volume in hippocampal subfields and decreased cortical thickness in regions supporting social cognition [19]. Longitudinal analyses demonstrate that persistent loneliness predicts progressive gray matter reduction in the posterior cingulate cortex and increased volume in the rostral anterior cingulate cortex, areas involved in emotional regulation and self-referential processing [37]. Functional connectivity patterns reveal reduced modularity within default and limbic networks among lonely individuals, potentially reflecting impaired integration of social information processing [13]. White matter microstructural analyses identify reduced fractional anisotropy in tracts connecting regions associated with empathy and self-efficacy, including the temporoparietal junction, anterior insula, and dorsolateral prefrontal cortex [15]. These structural alterations provide neural substrates for the cognitive biases characteristic of chronic loneliness, including heightened attention to social threat and negative social expectations [14].

### 3.3. Prefrontal Cortex Dysfunction and Social Cognition

The prefrontal cortex (PFC), comprising dorsolateral (dlPFC), ventrolateral (vlPFC), and ventromedial (vmPFC) subregions, plays central roles in cognitive control, emotion regulation, and social decision-making functions characteristically impaired in chronic loneliness [13,14,18]. Neuroimaging studies reveal that lonely individuals demonstrate altered PFC activation during social cognitive tasks, with reduced vmPFC activity during positive social evaluation and enhanced dlPFC recruitment during social threat processing, suggesting compensatory cognitive control attempts to manage negative social expectations [13,14].

The vmPFC specifically supports the valuation of social stimuli and the integration of affective information into decision-making processes [13,18]. Structural analyses demonstrate reduced gray matter volume in vmPFC among chronically lonely individuals, correlating with difficulties in social reward learning and flexible updating of social expectations [18,19,37]. The dlPFC, critical for executive functions and working memory, shows altered functional connectivity with limbic regions in loneliness, potentially underlying difficulties in emotion regulation and cognitive reappraisal of negative social experiences [13,14,15].

Prefrontal–limbic connectivity disruptions contribute to the maintenance of maladaptive cognitive biases in loneliness [13,14]. Reduced top-down prefrontal inhibition of amygdala responses produces sustained threat detection and difficulty disengaging from negative social cues [14]. These circuit-level alterations provide neural mechanisms for the cognitive–behavioral patterns perpetuating loneliness, including attention biases toward social threat, interpretation biases favoring negative social meanings, and memory biases toward rejection experiences [13,14].

### 3.4. Ventral Striatum, Nucleus Accumbens, and Social Reward Processing

The ventral striatum, particularly the nucleus accumbens (NAc), represents a critical node for reward processing and motivational drive toward social connection [1,17,20]. As detailed in Section 3.1, lonely individuals show paradoxical patterns of ventral striatal activation—increased responses when viewing close others, yet reduced motivation for actual social approach behaviors [13,17]. This apparent contradiction reflects complex alterations in reward circuit function, where social stimuli retain or even increase reward value representation, yet behavioral expression of social motivation becomes impaired [1,17,20].

Cell-type specific analyses reveal that chronic social isolation produces alterations in medium spiny neurons, the principal cell type in NAc, including changes in dendritic spine density and altered expression of dopamine and glutamate receptors [21,36]. These cellular adaptations modify the computational properties of NAc circuits, potentially explaining dissociations between wanting (motivational drive) and liking (hedonic experience) of social interaction [1,17]. Reduced motivation to engage socially despite intact or heightened social craving characterizes the behavioral phenotype of chronic loneliness [13,17].

Gene expression analyses in human NAc tissue reveal that loneliness is associated with altered transcription of genes involved in synaptic plasticity, inflammatory signaling, and neurodegenerative processes [20,42]. Overlap between loneliness-associated gene expression signatures and genetic risk factors for Alzheimer’s and Parkinson’s diseases suggests molecular convergences linking social disconnection to neurodegeneration risk, as discussed in Section 4.3 [20,42].

### 3.5. Amygdala Hyperreactivity and Threat Detection

The amygdala, essential for threat detection and emotional learning, demonstrates hyperreactivity in lonely individuals, particularly to negative social stimuli [13,14]. Functional neuroimaging studies consistently show enhanced amygdala activation when lonely participants view rejection-related stimuli, hostile faces, or ambiguous social cues capable of negative interpretation [13,14]. This heightened threat detection creates vigilance toward potential social danger, contributing to the hypervigilance characteristic of chronic loneliness [14].

Structural analyses reveal complex patterns of amygdala volume changes in loneliness, with some studies reporting increases potentially reflecting chronic activation-induced hypertrophy, while others find decreases suggesting atrophy from sustained stress exposure [18,19,37]. These discrepancies may reflect nonlinear temporal trajectories or individual differences in stress responsivity [32,37]. Amygdala functional connectivity patterns demonstrate alterations, with increased coupling to anterior insula (supporting interoceptive awareness of social pain) and reduced connectivity to prefrontal regulatory regions [13,15,18].

The amygdala’s central role in social threat detection extends to influencing memory consolidation, attention allocation, and physiological stress responses [9,14]. Enhanced amygdala reactivity in loneliness correlates with HPA axis activation and inflammatory markers, suggesting this brain region serves as a key node linking subjective social threat perception to systemic physiological consequences [9,27,31]. Interventions targeting amygdala reactivity, including mindfulness-based approaches and cognitive restructuring, show promise for reducing threat-focused processing patterns [43,44].

### 3.6. Anterior Insula and Social Pain Processing

The anterior insula serves critical functions in interoceptive awareness, the perception of internal bodily states, and in processing social pain, the distressing experience of social rejection or exclusion [13,15]. Neuroimaging studies demonstrate that social rejection activates the anterior insula in patterns overlapping with physical pain processing, supporting the concept that social pain recruits neural circuitry evolutionarily conserved for signaling threats to physical integrity [13]. In chronic loneliness, the anterior insula shows sustained hyperactivation during social evaluation contexts [13,15].

The insula’s role extends beyond pain processing to include integration of emotional, cognitive, and sensory information supporting subjective feeling states [13,18]. Altered insula function in loneliness contributes to the heightened sensitivity to rejection and social exclusion experiences that characterize this condition [13]. The anterior insula demonstrates altered connectivity with the amygdala (amplifying negative affect) and reduced connectivity with prefrontal regulatory regions (impairing emotion regulation) [13,15,18].

Interventions targeting interoceptive processes, including mindfulness meditation and body-focused therapies, may ameliorate loneliness through effects on insular cortex function [43,44]. These approaches train individuals to observe internal experiences non-judgmentally, potentially reducing the amplification of social pain signals characteristic of loneliness [44].

## 4. Developmental Trajectories and Critical Windows of Vulnerability

### 4.1. Lifespan Patterns: The U-Shaped Trajectory

The impact of social isolation and loneliness on neuroendocrine function varies substantially across the lifespan, with specific developmental periods conferring heightened vulnerability. Understanding these age-related differences is essential for identifying optimal intervention windows and developing targeted prevention strategies. Large-scale investigations reveal that loneliness follows a U-shaped pattern across the lifespan rather than a linear trajectory. A coordinated analysis of 128,118 participants (ages 13–103) across nine longitudinal studies from over 20 countries demonstrated elevated loneliness in young adulthood (peak age around 30), progressive decline through midlife (lowest at ages 40–50), and resurgence in older adulthood (peak > 80 years) [45]. This pattern remained consistent across diverse cultural contexts, suggesting fundamental developmental processes underlying age-related vulnerability.

The mechanisms driving this distribution reflect life-stage transitions. Young adulthood involves identity formation, partnership establishment, and career initiation—periods when failure to achieve normative milestones predicts heightened loneliness. Midlife protection derives from established social roles (employment, marriage, parenting) that inherently involve regular social contact. Late-life increases reflect accumulated losses: spousal death, retirement, mobility limitations, and social network contraction [46]. Critically, while population-level patterns show this U-shape, individual trajectories vary substantially, with approximately 50–60% maintaining stable low loneliness, 15–20% experiencing chronic high loneliness, and 10–15% showing increasing or decreasing patterns over time [47].

### 4.2. Adolescence as a Neurobiological Critical Period

Accumulating evidence supports the view that adolescence (ages 12–18) is a critical period during which social isolation exerts profound, potentially permanent neurobiological effects. Critical periods represent developmental windows when specific experiences disproportionately influence neural circuit organization, with effects persisting beyond the sensitive window [36].

The neurobiological foundation for adolescent vulnerability involves ongoing prefrontal cortex maturation, extensive synaptic pruning, progressive myelination, and dopaminergic system reorganization. Dopamine receptor expression undergoes dramatic changes during adolescence, with transient overproduction followed by pruning to adult levels—a process that may trigger critical period plasticity. Perturbations during this window, such as social isolation-induced VTA hyperexcitability, can permanently alter circuit function [36].

Experimental evidence from rodent models demonstrates that adolescent social isolation produces neurobiological changes persisting into adulthood even after normal housing restoration. These include reduced dendritic spine density, simplified dendritic arborization, decreased synaptophysin expression, and altered microtubule stability (reduced MAP-2) in prefrontal cortex [36]. Notably, monoamine oxidase inhibitor treatment during resocialization can reverse dendritic morphological deficits, indicating potential for pharmacological rescue even after developmental insults.

Human neuroimaging supports these findings. Analysis of 2809 youth (median age 12) revealed that socially withdrawn adolescents exhibited reduced gray matter volumes and altered connectivity in insula, anterior cingulate cortex, and superior temporal gyrus, with widespread alterations across default mode, salience, and frontoparietal networks [48]. Longitudinal studies show that adolescents experiencing persistent loneliness demonstrate elevated risk for mental health problems, lower educational attainment, and continued social difficulties years later, even when loneliness resolves—suggesting programming effects during this critical window [49].

### 4.3. Aging, Neurodegeneration, and Dementia Risk

Older adulthood represents a second vulnerability period, with severe consequences for cognitive health. Prospective studies consistently show loneliness predicts accelerated mental decline and incident dementia independent of objective isolation and established risk factors through mechanisms involving hippocampal atrophy and cortical thinning [16]. The Framingham Study (*n* = 2308, mean age 73) found baseline loneliness conferred a 54% increased 10-year dementia risk (HR = 1.54, 95% CI: 1.06–2.24) with age-dependent effects. Critically, this association showed age dependence: participants aged 60–79 exhibited a three-fold increased risk (HR = 3.03, 95% CI: 1.63–5.62) among *APOE ε4* non-carriers, whereas no association emerged in those ≥80 years, suggesting that younger-old adults represent the peak vulnerability [16,50]. Persistent loneliness predicts longitudinal deterioration in multiple cognitive domains, including reasoning, verbal memory, and executive function, independent of objective social isolation [15]. Importantly, recovery from loneliness (transient loneliness) appears protective against brain structure alterations, suggesting potential reversibility of neural changes [15].

The relationship between loneliness and cognitive function exhibits domain specificity, with executive function and visuospatial abilities showing greater vulnerability than episodic memory, distinguishing loneliness-associated cognitive impairment from Alzheimer’s disease pathology [51]. Social isolation contributes to hippocampal volume reduction and cortical thinning within a population-based longitudinal study, with within-subject effects comparable to the between-subject impacts, indicating modifiable risk [37].

Co-expression network analysis identified several differentially expressed genes in the nucleus accumbens of lonely individuals that overlap with known Alzheimer’s disease risk loci [20]. These loneliness-associated genes include *NECTIN2*, which functions in synaptic organization and cell adhesion at pre- and postsynaptic sites; disruption of NECTIN2 may compromise synaptic integrity, contributing to early Alzheimer’s pathology [52,53]. *GPNMB* is expressed by activated microglia and is involved in phagocytosis of pathological material; elevated GPNMB levels correlate with disease severity in Parkinson’s disease and lysosomal dysfunction in neurodegeneration [54,55]. *HLA-DRB5*, a major histocompatibility complex class II gene, is expressed on reactive microglia in Alzheimer’s and Parkinson’s disease brains; upregulation of *HLA-DR* reflects inflammatory activation and aberrant microglial responses that may contribute to synaptic dysfunction [56,57]. *NPAS3* regulates adult hippocampal neurogenesis and exhibits circadian-dependent transcriptional activity; dysfunction of *NPAS3* impairs neurogenesis in the hippocampal subgranular zone and has been linked to psychiatric disorders [58,59]. Collectively, these gene expression changes in loneliness may create a pro-neurodegenerative molecular environment characterized by synaptic vulnerability, microglial dysfunction, and impaired neurogenic capacity—mechanisms that intersect with established Alzheimer’s disease pathways.

## 5. Sex Differences in Neuroendocrine Responses to Social Isolation

### 5.1. Hormonal Regulation of Sex-Specific Outcomes

Substantial evidence indicates sex differences in neuroendocrine responses to social isolation, mediated by gonadal hormones and sex chromosome effects [39]. Female rodents demonstrate greater vulnerability to chronic adolescent stress-induced depressive behaviors compared to males, accompanied by distinct patterns of glucocorticoid receptor signaling in the hippocampus [32]. These sex-specific outcomes persist into adulthood, suggesting developmental programming of stress responsivity [32]. Social exclusion paradigms in humans reveal gender-specific hormonal responses, with testosterone decreasing following exclusion in both sexes, while progesterone increases selectively in females [38]. These sex hormone fluctuations may reflect differential coping strategies or distinct neuroendocrine adaptations to social stress [38]. Older women report higher baseline loneliness than men, yet demonstrate differential cortisol associations, with social loneliness predicting elevated morning cortisol specifically in men [25].

### 5.2. Circuit-Specific Sex Differences

AVP and oxytocin systems exhibit pronounced sexual dimorphism in anatomical distribution and functional roles [39]. Male rats possess greater AVP-immunoreactive cell numbers in the lateral septum and posterior bed nucleus of stria terminalis (BST) compared to females, correlating with sex differences in anxiety-related and social behaviors [39]. These neuroanatomical sex differences arise from organizational effects of perinatal androgens on brain development [40]. Molecular analyses in VTA reveal sex-specific transcriptional responses to chronic social isolation, with females showing downregulation of hypocretin/orexin, a neuropeptide regulating arousal and reward [41]. Orexin-A treatment rescues social withdrawal specifically in isolated females, identifying sex-selective therapeutic targets [41]. Males demonstrate greater reliance on alcohol consumption as a coping mechanism for loneliness, though this association depends on measurement precision [60]. These sex differences in neuroendocrine and behavioral responses to social isolation are summarized in Table 1.

## 6. Discussion

This review synthesizes evidence demonstrating that social isolation and loneliness function as potent chronic stressors that produce widespread neuroendocrine dysregulation with consequent behavioral and health impacts. The integration of molecular, circuit, and systems-level analyses reveals multifaceted biological pathways linking perceived and objective social disconnection to adverse outcomes across the lifespan. Understanding these mechanisms offers opportunities to develop targeted interventions for this growing public health challenge.

### 6.1. Neuroendocrine Pathways: From Mechanism to Intervention

The HPA axis represents a central mediator of social isolation effects, with chronic activation leading to altered diurnal cortisol rhythms, glucocorticoid resistance, and downstream effects on immune function and metabolism [7]. This dysregulation manifests through flattened cortisol slopes, elevated awakening responses, and increased total daily cortisol output [25]. Paradoxically, chronic loneliness induces glucocorticoid resistance at the cellular level, wherein target tissues become less responsive to cortisol signaling despite elevated circulating levels [32].

Importantly, the nature of HPA axis dysregulation appears to differ fundamentally depending on the developmental timing of isolation exposure. Social isolation during adolescence produces lasting “programming” effects on stress responsiveness that persist into adulthood, even after resocialization, despite often showing minimal impact on basal HPA axis function [39]. When corticosterone concentrations were measured longitudinally in adolescent-isolated mice, they displayed glucocorticoid insufficiency rather than elevated tone during the isolation period, which normalized by adulthood [61]. However, stress reactivity assessments reveal sex-specific alterations, with previously isolated males exhibiting diminished corticosterone responses to acute stressors while isolated females mount more robust responses [47]. These findings suggest that adolescent isolation fundamentally reprograms stress response systems during a critical neurodevelopmental window through mechanisms involving altered glucocorticoid receptor–FKBP5 interactions [15,32]. In contrast, chronic loneliness in older adulthood produces distinct HPA dysregulation characterized by sustained basal hypercortisolemia and altered diurnal patterns [12,25], reflecting accumulated allostatic load rather than developmental programming. This distinction has critical implications for intervention timing and approach: adolescent exposure may require interventions targeting developmental plasticity mechanisms during or shortly after the critical period, whereas aging-related dysregulation may respond to interventions addressing chronic stress and glucocorticoid resistance.

Proinflammatory gene expression upregulation, orchestrated through the conserved transcriptional response to adversity (CTRA), provides a molecular mechanism linking loneliness to cardiovascular disease, neurodegeneration, and accelerated aging [29]. The CTRA represents an evolutionary adaptation prioritizing innate immune responses while suppressing adaptive immunity, potentially reflecting defensive biological strategies against wounding risk during social conflict [28].

Neuropeptide systems, particularly oxytocin and vasopressin, offer promising therapeutic targets for social disconnection [7]. Social isolation alters oxytocinergic signaling within mesocorticolimbic circuits, with reduced oxytocin receptor binding in regions mediating social reward [34]. Intranasal oxytocin administration shows potential for reducing loneliness, though individual variation in baseline bonding behaviors moderates treatment responsiveness [22].

A persistent controversy concerns the relative contributions of subjective loneliness versus objective social isolation to these neuroendocrine outcomes [5]. Outcome-wide longitudinal approaches suggest differential associations, with loneliness more strongly predicting mental health outcomes while isolation correlates with physical health measures. The confluence of high loneliness and high isolation produces amplified effects compared to either condition alone, highlighting the necessity of assessing both constructs concurrently [16].

Establishing causal relationships between loneliness and biological outcomes presents methodological challenges [62]. Mendelian randomization analyses reveal that genetic predisposition to loneliness causally influences only a subset of health conditions, suggesting loneliness may function more as a surrogate marker than a direct cause for many diseases, with bidirectional relationships complicating causal inference [62].

Future research should leverage advances in molecular profiling to identify biological subtypes of loneliness with distinct neuroendocrine signatures [29]. Epigenetic modifications, including DNA methylation and histone acetylation changes in stress-responsive and immune genes, represent promising mechanisms linking loneliness to long-term health outcomes [63]. Personalized interventions guided by individual neuroendocrine profiles may improve treatment efficacy [63].

### 6.2. Neural Circuit Adaptations and Behavioral Manifestations

Loneliness profoundly affects mesocorticolimbic reward circuits comprising the ventral tegmental area, nucleus accumbens, and prefrontal cortex [17]. Functional neuroimaging studies reveal that lonelier individuals exhibit increased ventral striatal activation when viewing close others, suggesting heightened reward value attribution to social reconnection opportunities. This “yearning for connection” reflects adaptive motivation to restore social bonds, yet chronic loneliness paradoxically impairs social approach behaviors through maladaptive cognitive processes [13].

Large-scale neuroimaging studies reveal that loneliness is associated with altered structural and functional connectivity within the default mode network, comprising the medial prefrontal cortex, posterior cingulate cortex, and hippocampal formation [18]. Functional connectivity patterns reveal reduced modularity within default and limbic networks among lonely individuals, potentially reflecting impaired integration of social information processing [13]. White matter microstructural analyses identify reduced fractional anisotropy in tracts connecting regions associated with empathy and self-efficacy [15]. These structural alterations provide neural substrates for the cognitive biases characteristic of chronic loneliness, including heightened attention to social threat and negative social expectations.

The relationship between loneliness and cognitive function exhibits domain specificity, with executive function and visuospatial abilities showing greater vulnerability than episodic memory, distinguishing loneliness-associated cognitive impairment from Alzheimer’s disease pathology [50]. Gene expression analyses in human nucleus accumbens reveal that loneliness-associated transcriptional changes overlap substantially with genetic risk factors for Alzheimer’s and Parkinson’s diseases [42]. These molecular convergences provide mechanistic explanations for epidemiological associations between loneliness and neurodegenerative disease prevalence.

Current interventions for loneliness demonstrate modest effectiveness, with considerable heterogeneity in approaches and outcomes [43]. Cognitive–behavioral therapies targeting maladaptive social cognitions show promise by addressing negative expectations and attentional biases that perpetuate loneliness [44]. Whether psychological interventions normalize HPA axis function, reverse CTRA gene expression patterns, or restore neural circuit function requires systematic investigation.

Emerging technologies, including optogenetics, chemogenetics, and circuit-specific recordings, enable precise interrogation of neural pathways mediating loneliness-associated behaviors [20]. Future studies should characterize molecular markers distinguishing functionally discrete neuronal subtypes and map their synaptic connectivity patterns [64]. Translational applications may include non-invasive neuromodulation techniques targeting circuits implicated in social cognition and reward processing [65].

### 6.3. Interaction Between Developmental Timing and Sex Differences

The intersection of developmental stage and biological sex represents a critical yet understudied dimension in understanding vulnerability to social isolation. Adolescence constitutes a neurobiological critical period characterized by ongoing prefrontal cortex maturation, extensive synaptic pruning, progressive myelination, and dopaminergic system reorganization. During this sensitive developmental window, sex-specific hormonal environments differentially sculpt neural circuit organization and stress responsivity. Accumulating evidence from rodent models demonstrates that chronic adolescent social isolation produces sexually dimorphic behavioral and neurobiological consequences that persist into adulthood, even after resocialization [48]. Male rodents exposed to adolescent isolation exhibit heightened aggression toward intruders and altered prefrontal cortex-to-basolateral amygdala connectivity, while isolated females display increased social withdrawal behaviors linked to prefrontal cortex-to-ventral tegmental area pathway dysfunction [48]. These divergent outcomes suggest that gonadal hormones and sex-chromosome effects interact with critical-period plasticity mechanisms to determine which neural circuits are most vulnerable to isolation stress.

The molecular mechanisms underlying sex-specific developmental vulnerability involve differential regulation of stress-responsive gene expression programs. Female rodents exposed to chronic adolescent stress exhibit elevated hippocampal glucocorticoid receptor-FKBP5 interactions following acute stress challenges in adulthood, potentially contributing to impaired stress recovery and increased vulnerability to affective disorders [32]. In contrast, male rodents show region-specific upregulation of genes in the basolateral amygdala under transcriptional control of activated transcription factor CREB, with CREB inhibition normalizing gene expression and reversing aggressive behaviors [48]. The neuropeptide systems also demonstrate pronounced sexual dimorphism, with vasopressin and oxytocin systems regulating social behaviors in sex-specific ways during development, with perturbations potentially causing one sex to be more vulnerable and the other more resilient to specific social and emotional disorders [46].

Human longitudinal studies provide convergent evidence for sex-specific developmental trajectories of loneliness vulnerability. The timing of isolation exposure critically determines reversibility potential, with social isolation limited to specific early adolescent sub-periods producing lasting deficits, whereas later isolation produces no enduring effects, suggesting the existence of particularly vulnerable developmental windows [39]. Furthermore, the mode of resocialization following juvenile isolation significantly impacts neural circuit recovery, with gradual reintegration showing greater efficacy than abrupt transitions in restoring prefrontal cortex myelination and function [66].

The intergenerational dimension adds complexity to sex-specific developmental vulnerability. Parental chronic stress and social isolation predict offspring HPA axis function, inflammatory profiles, and social behavior patterns through multiple mechanisms, including epigenetic modifications (DNA methylation patterns in glucocorticoid receptor genes), altered maternal care behaviors affecting offspring stress responsivity, and potential germline transmission of stress-induced epigenetic marks [64]. The FKBP5 gene, which modulates glucocorticoid receptor sensitivity and is altered in lonely individuals, demonstrates intergenerational epigenetic transmission in both animal models and in humans exposed to early-life adversity. Understanding whether loneliness-specific biological signatures transfer across generations could inform preventive interventions targeting at-risk families. Whether these signatures transfer in sex-specific patterns—potentially explaining observed sex differences in depression and anxiety prevalence—represents a critical gap requiring investigation through longitudinal multigenerational studies with sex-stratified analyses.

### 6.4. Temporal Patterns of Isolation: Intermittent vs. Chronic Effects

The temporal dynamics of social isolation exposure profoundly influence neuroendocrine and behavioral outcomes, yet most research has focused on continuous chronic isolation paradigms rather than intermittent patterns that may better model real-world human experiences. Accumulating evidence suggests that continuous versus intermittent isolation produces distinct neurobiological signatures with differing potential for reversibility. Rodent studies employing varying isolation durations reveal dose-dependent effects, with longer isolation periods producing more severe and persistent deficits. Studies utilizing 10–14 week isolation periods demonstrate more pronounced anxiety-like behaviors and cognitive deficits compared to shorter 30-day exposures, with some deficits proving resistant to pharmacological intervention [39]. This duration dependence suggests progressive neuroadaptations accumulate under sustained isolation, potentially crossing thresholds beyond which spontaneous recovery becomes limited.

The concept of critical period closure further complicates temporal pattern effects. Social isolation during adolescence produces neurobiological changes persisting into adulthood, even after normal housing restoration, including reduced dendritic spine density, simplified dendritic arborization, decreased synaptophysin expression, and altered microtubule stability in the prefrontal cortex [36]. Notably, monoamine oxidase inhibitor treatment during resocialization can reverse dendritic morphological deficits, indicating that pharmacological rescue remains possible even after developmental insults, though the therapeutic window narrows as critical periods close [36].

The reversibility potential appears to depend on when the intervention occurs relative to the developmental stage. Adolescent enrichment can partially reverse the social isolation syndrome, though the reversal remains incomplete even when enrichment is provided during adolescence [67]. The extent to which interventions applied after critical period closure can restore function remains to be investigated.

Emerging evidence indicates that intermittent social isolation produces unique neurobiological effects distinct from continuous exposure. Recent studies demonstrate that ISI in adult male mice enhances social investigation behavior reminiscent of craving-like states, while paradoxically impairing social recognition memory [47]. This dissociation between social motivation and memory suggests distinct neural circuit adaptations compared to continuous isolation paradigms.

Intermittent isolation paradigms activate specific brain regions including the paraventricular nucleus of the hypothalamus, lateral septum, and thalamic periventricular gray, with effects that are selective for social domains without affecting anxiety-like behaviors or spatial memory [47]. Intermittent social isolation produces selective cognitive deficits, specifically impairing spatial learning in the Morris water maze while sparing general locomotor function. These behavioral changes correlate with reduced parvalbumin-positive interneurons in hippocampal CA1 and dentate gyrus, suggesting that even intermittent isolation is sufficient to alter inhibitory circuit function in memory-related brain regions [68].

This pattern suggests that intermittent or varied stressor exposure during development may activate stress inoculation mechanisms, potentially through enhanced glucocorticoid receptor regulation or altered HPA axis feedback sensitivity. Human data from the COVID-19 pandemic provides natural experiment conditions to examine intermittent isolation effects. Forced social isolation during lockdowns intensified the positive association between momentary loneliness and salivary cortisol levels, suggesting context-dependent modulation of neuroendocrine stress responses [6]. Importantly, individuals experiencing intermittent loneliness (only at age 12 or only at age 18) showed differential outcomes compared to those with recurrent loneliness across both time points, with early adolescent loneliness having specific implications for educational attainment independent of recurrence [41].

The relationship between isolation chronicity and inflammatory system activation presents another critical temporal dimension. Chronic loneliness produces sustained alterations in the conserved transcriptional response to adversity, characterized by upregulated expression of proinflammatory genes and downregulated antiviral genes [2]. Whether intermittent loneliness triggers similar CTRA patterns or whether periodic social reconnection opportunities prevent this transcriptional reprogramming remains unknown. Longitudinal studies tracking individuals across varying patterns of social connection and isolation with repeated biological assessments could illuminate whether intermittent isolation produces cumulative effects approximating chronic exposure or whether periods of social reintegration enable biological recovery. Such investigations hold particular relevance for designing effective interventions, as they would inform whether brief targeted social connection interventions can interrupt pathological trajectories established by previous isolation experiences, or whether sustained long-term reintegration is necessary to normalize neuroendocrine function and reverse inflammatory gene expression patterns [1,44].

### 6.5. Lifestyle Interventions: Potential Therapeutic Targets

Lifestyle factors represent modifiable intervention targets that operate bidirectionally with loneliness, offering multiple points for therapeutic intervention. Understanding these relationships provides opportunities for comprehensive approaches that simultaneously address neuroendocrine dysregulation and promote social reconnection.

Physical exercise demonstrates particular promise as a multi-mechanistic intervention. Exercise interventions reduce loneliness through increased social interaction opportunities in group settings, improved mood via endorphin release, and reduced inflammatory markers, and enhanced neuroplasticity supporting cognitive function [61]. Lonely individuals demonstrate reduced physical activity engagement, potentially through motivation deficits linked to reward circuit dysfunction, while physical inactivity independently predicts increased loneliness over time [69,70]. These bidirectional effects suggest that structured exercise programs, particularly those with social components, could interrupt self-perpetuating cycles of inactivity and isolation.

Sleep quality interventions warrant prioritization given reciprocal relationships between loneliness and sleep disturbances. Lonely individuals report poorer sleep quality, difficulty initiating sleep, and reduced sleep efficiency, mediated by heightened threat vigilance, altered circadian rhythms from irregular social zeitgebers, and inflammatory activation [71,72,73,74]. Critically, experimental sleep deprivation directly increases feelings of loneliness and social withdrawal, suggesting that sleep restoration interventions could improve social motivation and perception. Addressing sleep disturbances may also interrupt cascading effects on HPA axis dysregulation and inflammatory activation.

Dietary and nutritional interventions represent underexplored but potentially valuable targets. Social isolation influences eating behaviors, with solitary eating associated with less nutritious food choices and deficient intake of key micronutrients including magnesium, potassium, vitamin B6, folate, and vitamin C [75,76,77]. Interventions promoting communal eating or addressing nutritional deficiencies could simultaneously improve physiological health and provide social connection opportunities. Similarly, targeted interventions for substance use are critical given that loneliness predicts increased alcohol and drug consumption as coping mechanisms, which subsequently impair social functioning and create vicious cycles [78,79,80,81].

Technology-based interventions show mixed efficacy. While social media platforms offer connection opportunities, particularly for geographically isolated individuals, passive consumption may increase loneliness through social comparison processes [82,83,84,85]. Future interventions should focus on promoting active engagement and meaningful online interactions while limiting passive scrolling. Comprehensive multi-component interventions targeting multiple lifestyle factors simultaneously may prove most effective, though this hypothesis requires systematic investigation through well-controlled trials with biological outcome measures including cortisol patterns, inflammatory markers, and neural circuit function.

### 6.6. Limitations and Methodological Considerations

Several limitations constrain current understanding of neuroendocrine and behavioral consequences of social isolation and loneliness, highlighting priorities for future research.

Inconsistency in loneliness assessment across studies limits direct comparability and meta-analytic synthesis. Multiple loneliness scales exist (UCLA Loneliness Scale versions 1–3, De Jong Gierveld Loneliness Scale, single-item measures), each capturing potentially different constructs or emphasizing different loneliness dimensions (social vs. emotional; romantic vs. family vs. friend loneliness).

Most evidence linking loneliness to neuroendocrine and health outcomes derives from observational longitudinal or cross-sectional studies that cannot definitively establish causality. While longitudinal designs with loneliness predicting subsequent biological changes support causal hypotheses, reverse causation and residual confounding remain possible. Mendelian randomization studies provide genetic instruments for causal inference but capture only the variance explained by common genetic variants and may not reflect the effects of environmental exposures.

Most research on loneliness biology originates from Western, educated, industrialized, rich, and democratic populations, limiting generalizability to diverse cultural contexts where meanings of social connection differ. Cultural variations in individualism–collectivism, multigenerational household norms, and acceptable solitude levels may moderate relationships between loneliness and health.

Rodent models of social isolation provide powerful tools for mechanistic investigation but face inherent limitations. Species differences in social systems, developmental trajectories, and brain organization constrain direct translation. Laboratory housing conditions may not adequately model human social complexity. Most animal studies employ complete isolation rather than the partial social contact characterizing human loneliness.

While numerous interventions targeting loneliness exist, rigorous evidence for their efficacy in normalizing neuroendocrine function remains limited. Most intervention studies assess subjective loneliness or related mental health outcomes but rarely include biological measures. When biological outcomes are assessed, follow-up periods often are insufficient to detect sustained changes in inflammatory markers, cortisol patterns, or neural structure.

## 7. Conclusions

The integration of social genomics, advanced neuroimaging, circuit-based approaches, and clinical intervention research positions behavioral endocrinology to make substantial contributions toward addressing the loneliness epidemic. Understanding hormonal and behavioral consequences of social disconnection not only illuminates fundamental principles of social neuroscience but also informs the development of targeted interventions to improve health and well-being across populations.

Critical remaining research gaps include a mechanistic understanding of epigenetic modifications that mediate the long-term consequences of social isolation, optimal timing and content of interventions across the lifespan, and the translation of preclinical findings into clinical applications. The field would benefit from standardized assessment tools, longitudinal study designs with comprehensive biological measures, and further integration of molecular, circuit, and systems-level analyses. As social disconnection continues to represent a major public health challenge, particularly following global events like the COVID-19 pandemic, translating our growing mechanistic understanding into effective interventions remains a critical research priority with profound implications for individual and population health.

## Figures and Tables

**Table 1:** Sex Differences in Neuroendocrine and Behavioral Responses to Social Isolation.

Parameter	Female Response	Male Response	Significance/Implications	Citations
HPA Axis Reactivity	Greater vulnerability to chronic adolescent stress-induced depressive behaviors	More resilient to adolescent stress-induced depression	Sex-specific developmental programming of stress responsivity	[32]
GR Signaling	Elevated hippocampal GR-FKBP5 interactions following stress in adulthood	Less pronounced GR-FKBP5 interactions	Potentially contributes to impaired stress recovery in females	[32]
Sex Hormone Response to Social Exclusion	Progesterone increased (both exclusion/inclusion); testosterone decreased (exclusion)	Testosterone decreased (exclusion), increased (inclusion)	May reflect differential coping strategies	[38]
Cortisol Response	No significant association between loneliness and cortisol indices	Social and family loneliness predict elevated awakening cortisol	Sex-specific HPA axis responses to loneliness in older adults	[25]
Neuropeptide Systems	Lower baseline AVP	Greater AVP-immunoreactive cells in BST; higher baseline AVP	Organizational effects of perinatal androgens	[39]
Transcriptional Responses (VTA)	Downregulation of hypocretin/orexin	Normal hypocretin/orexin levels	Sex-selective therapeutic targets (orexin-A treatment rescues social withdrawal specifically in females)	[41]
Behavioral Coping	More likely to seek social support	Greater reliance on substance use (e.g., alcohol consumption)	Interventions may need to target different coping mechanisms	[60]
Prevalence	Higher romantic loneliness in older women; similar social and family loneliness	Lower reported loneliness but potentially higher mortality impact	Differential assessment needs and intervention approaches; type-specific loneliness varies by sex	[25,60]

BST = bed nucleus of stria terminalis; GR = glucocorticoid receptor; FKBP5 = FK506 binding protein 5; VTA = ventral tegmental area.

## Data Availability

No new data were created or analyzed in this study. Data sharing is not applicable to this article.

## Abbreviations

AMPA	α-amino-3-hydroxy-5-methyl-4-isoxazolepropionic acid
AP-1	activator protein-1
APOE	apolipoprotein E
AVP	arginine vasopressin
BCAM	basal cell adhesion molecule
BST	bed nucleus of stria terminalis
CTRA	conserved transcriptional response to adversity
FKBP5	FK506 binding protein 5
GPNMB	glycoprotein nonmetastatic melanoma protein B
GR	glucocorticoid receptor
HLA-DRB5	major histocompatibility complex, class II, DR beta 5
HPA	hypothalamic–pituitary–adrenal
IL-6	interleukin-6
MAP-2	microtubule-associated protein 2
NECTIN2	nectin cell adhesion molecule 2
NF-κB	nuclear factor kappa B
NPAS3	neuronal PAS domain protein 3
PVN	paraventricular nucleus
TNF-α	tumor necrosis factor-alpha
V1a	vasopressin 1a receptor
V1b	vasopressin 1b receptor
VTA	ventral tegmental area
